# Using real-time impedance-based assays to monitor the effects of fibroblast-derived media on the adhesion, proliferation, migration and invasion of colon cancer cells

**DOI:** 10.1042/BSR20140031

**Published:** 2014-07-29

**Authors:** Catríona M. Dowling, Carmen Herranz Ors, Patrick A. Kiely

**Affiliations:** *Department of Life Sciences, and Materials and Surface Science Institute, University of Limerick, Limerick, Ireland; †Stokes Institute, University of Limerick, Limerick, Ireland

**Keywords:** cancer progression, colon cancer, real-time cell analysis, stromal environment, xCELLigence, CAF, cancer-activated fibroblast, CI, cell index, DMEM, Dulbecco’s modified essential medium, ECM, extracellular matrix, FAK, focal adhesion kinase, HCTM, media derived from HCT116 cultures, HDF, human dermal fibroblast, HDFM, human dermal fibroblast medium, LC, lower chamber, MMP, matrix metalloproteinase, PDGF, platelet-derived growth factor, RTCA, real-time cell analysis, UC, upper chamber

## Abstract

Increasing our knowledge of the mechanisms regulating cell proliferation, migration and invasion are central to understanding tumour progression and metastasis. The local tumour microenvironment contributes to the transformed phenotype in cancer by providing specific environmental cues that alter the cells behaviour and promotes metastasis. Fibroblasts have a strong association with cancer and in recent times there has been some emphasis in designing novel therapeutic strategies that alter fibroblast behaviour in the tumour microenvironment. Fibroblasts produce growth factors, chemokines and many of the proteins laid down in the ECM (extracellular matrix) that promote angiogenesis, inflammation and tumour progression. In this study, we use a label-free RTCA (real-time cell analysis) platform (xCELLigence) to investigate how media derived from human fibroblasts alters cancer cell behaviour. We used a series of complimentary and novel experimental approaches to show HCT116 cells adhere, proliferate and migrate significantly faster in the presence of media from human fibroblasts. As well as this, we used the xCELLigence CIM-plates system to show that HCT116 cells invade matrigel layers aggressively when migrating towards media derived from human fibroblasts. These data strongly suggest that fibroblasts have the ability to increase the migratory and invasive properties of HCT116 cells. This is the first study that provides real-time data on fibroblast-mediated migration and invasion kinetics of colon cancer cells.

## Abstract 2

Here, we use an RTCA platform to assess the kinetics of colon cancer cell adhesion, proliferation, migration and invasion in response to media derived from fibroblasts. This work highlights the importance of considering the stromal environment in cancer treatment.

## INTRODUCTION

Carcinomas, the most common form of human cancer, are caused by the accumulation of somatic mutations in epithelial cells [1]. However, the development of a carcinoma is influenced heavily by the tumour microenvironment surrounding these epithelial cells [2]. This tumour stroma is dynamic and responsive and provides a permissive and supportive environment for tumour progression [3]. The interaction between the cancer cells and the stromal environment is ‘two way’, as cancer cells may trigger a reactive response in the stroma, while the stromal cells in the surrounding microenvironment can also directly affect the cancer cell responses [4].

Cancer cells produce a range of growth factors and proteases that modify their stromal environment. These include bFGF (basic fibroblast growth factor) [5], members of the VEGF (vascular endothelial growth factor) family [6], PDGF (platelet-derived growth factor) [7], EGFR (epidermal growth factor receptor) ligands [8], TGFβ (transforming growth factor-β) [9,10], among others. These components disrupt normal tissue homoeostasis and act to induce angiogenesis and promote the activation of surrounding stromal fibroblasts [11–13].

The tumour stroma includes a variety of non-epithelial cells for example inflammatory cells (lymphocytes, macrophages and mast cells) and fibroblasts along with a series of ECM (extracellular matrix) proteins and extracellular molecules [14,15]. These cells are not malignant, however, they acquire an abnormal phenotype and an altered function due to their interaction with the cancer cells [16]. It is becoming increasingly clear that fibroblasts are important in the development of carcinomas. The tumour stroma contains CAFs (cancer-activated fibroblasts), similar to the transformed fibroblasts observed in wound healing and fibrosis [16], that play a multifaceted role in tumour progression. The signals that mediate the transition of a normal fibroblast into a CAF are not fully understood. Activated fibroblasts found in different cancers are highly heterogeneous and are derived from various origins [17]. They inhibit early stages of tumour progression [18] but later become activated by several tumour-secreted factors and promote both tumour growth and progression [19]. CAFs produce αSMA, desmin, Thy-1 and FSP (fibroblast-specific protein), and overexpress PDGF receptors-β [19]. The tumour microenvironment can provide a selective pressure on the stromal environment, encouraging the expression of cell lineages carrying mutations. Normal fibroblasts can become CAFs through genetic and epigenetic alterations, through LOH (loss of heterozygosity), and dysregulation of the genes encoding oncogenes and tumour suppressors [20].

Activated fibroblasts promote tumour progression in several ways. They induce cancer cell invasion through cell–cell contacts and paracrine diffusible factors [21]. They secrete ECM components such as tenascin C and collagen types I and III to alter the tumour microenvironment [22]. Activated fibroblasts up-regulate the expression of serine proteases and MMPs (matrix metalloproteinases) such as uPA (urokinase-type plasminogen activator), MMP1, MMP2, MMP3 and MMP9, that degrade and remodel the ECM [23,24]. Cancer cells also generate proteolytic enzymes which remodel the ECM to facilitate a pro-migratory and pro-invasive environment [25]. Cross-talk between the cancer cells and activated fibroblasts converge to modify the adjacent ECM and to promote the transformed phenotype in cancer.

The scientific community have made significant advances in understanding the signalling and mechanical mechanisms underlying cell adhesion, cell spreading and migration in cancer. Understanding these mechanisms is helping us acquire a better appreciation of the diseased state. However, the tools we employ remain dependent on the use of labels or are limited to endpoint analysis. Recently, we have seen the emergence of RTCA (real-time cell analysis) platforms which facilitate both label free and operator independent investigation of cell behaviour by monitoring the cells in physiologically relevant conditions [26,27]. Here, we describe the use of the xCELLigence RTCA platform to capture real-time kinetic data. This system facilitates a more accurate characterization of colon cancer cells adhesion, migration and invasion as the cells respond to signals coming from HDFs (human dermal fibroblasts). This is the first study of its kind documenting the effects of fibroblasts on colon cancer cells using RTCA platforms and our results demonstrate that media derived from HDFs promotes the transformed phenotype.

## MATERIALS AND METHODS

### Cell culture

HCT116 colon cancer cell line was obtained from ATCC and the HDF cell line was originally obtained from invitrogen. These cell lines were cultured in complete DMEM (Dulbecco's modified essential medium) supplemented with 10% (v/v) of foetal bovine serum, 1% (v/v) of penicillin/streptomycin and 1% of L-glutamine. All cells were incubated at 37°C in a humidified 95% (v/v) air/5% (v/v) CO_2_ environment and were grown to 50–80% confluence. Cellular suspensions were obtained by adding 0.5% trypsin to the cultures and incubating at 37°C at 5% CO_2_.

### Preparation of fibroblast-derived media

24 h prior to conducting experiments, HCT116 and HDF cells were trypsinized. 50 000 cells/ml of each cell line were cultured in 75 cm tissue culture flasks with DMEM. Media was removed from these cultures and used for the experiments described in the subsequent sections.

### Monitoring cell adherence and proliferation

Cell adherence and proliferation was monitored in real-time using the xCELLigence system E-Plate. 40 000 HCT116 cells were seeded in each well. Media taken from, HDF cultures, HCT116 cultures (prepared as described above) or fresh DMEM was added to the well. The impedance value of each well was automatically monitored by the xCELLigence system for duration of 24 h and expressed as a CI (cell index) value. Data for cell adherence were normalized at 40 min. Normalized CI is calculated by dividing CI at the normalized time into the original CI. The rate of cell growth was determined by calculating the slope of the line between two given time points.

### Cell adhesion assay

HCT116 cells were seeded in a 96-well plate and allowed to attach for indicated times at 37°C. Unbound cells were removed by inverting and gentle washing in PBS before cells were fixed with methanol at −20°C for 5 min. Cells were stained with 0.1% crystal violet and measured by reading the absorbance at 595 nm. Cells were also seeded in a 96-well plate and photographed at the indicated times.

### Co-culturing HDF cells and HCT116 cells

The rate of cell proliferation was monitored in real time using the xCELLigence system (E-plate and E-insert). Totally 40000 HCT116 cells were seeded in the E-plate 16, 24 h later an E-plate insert containing HDF cells was placed into the E-plate 16. Data were normalized at 24 h and the impedance value was automatically monitored by the xCELLigence system for 72 h and expressed as a CI value.

### Monitoring cell migration

The rate of cell migration was monitored in real-time with the xCELLigence system (CIM-plates). Approximately 4 h prior to conducting the experiment HCT116 cells were serum starved. The UC (upper chamber) of the CIM-plates was coated with 1μg/μl of fibronectin. A total of 40 000 HCT116 cells were seeded in each well of the UC in serum-free media. Media taken from either, HDF cultures, HCT116 cultures or fresh DMEM was added to each well of the LC (lower chamber). The CIM-plates was left in an incubator for 1 h to allow cell attachment. The impedance value of each well was automatically monitored by the xCELLigence system for duration of 48 h and expressed as a CI value.

### Monitoring cell invasion

The rate of cell invasion was monitored in real-time with the xCELLigence system CIM-plates. 4 h prior to conducting the experiment HCT116 cells were serum starved. The UC of the CIM-plates was coated with 1 μg/μl of fibronectin and a 1:40 solution of Matrigel™ (BD Biosciences). A total of 40 000 HCT116 cells were seeded in each well of the UC in serum-free media. Media taken from, HDF cultures, HCT116 cultures or fresh DMEM was added to each well of the LC. The CIM-plates was left in an incubator for 1 h to allow cell attachment. The impedance value of each well was automatically monitored by the xCELLigence system for duration of 24 h and expressed as a CI value.

### Statistical analysis

Statistical analyses were performed using the SPSS 20 Statistical Package. Differences between groups were calculated using Welch Anova and multiple comparisons were performed using Bonferroni Correction. A *P* value of <0.05 was considered statistically significant.

## RESULTS

### Monitoring cell behaviour in real-time

The xCELLigence system is a label-free cell-based assay system integrating microelectronics and cell biology and is suitable for uninterrupted monitoring of biological processes of living cells. It uses specially designed microtitre plates containing interdigitated gold microelectrodes to non-invasively monitor the viability of cultured cells. The electrodes measure the electrical impedance of the cell population in each well and it provides quantitative real-time information about the status of the cells. The continuous monitoring of cell viability by the xCELLigence system makes it possible to distinguish between different perturbations, such as proliferation, migration and invasion [28]. Recently, this platform has proved very informative in monitoring the toxicity of compounds [29], biomaterials [30], inhibitors [31] and the cell differentiation process [32,33].

**Figure 1:**
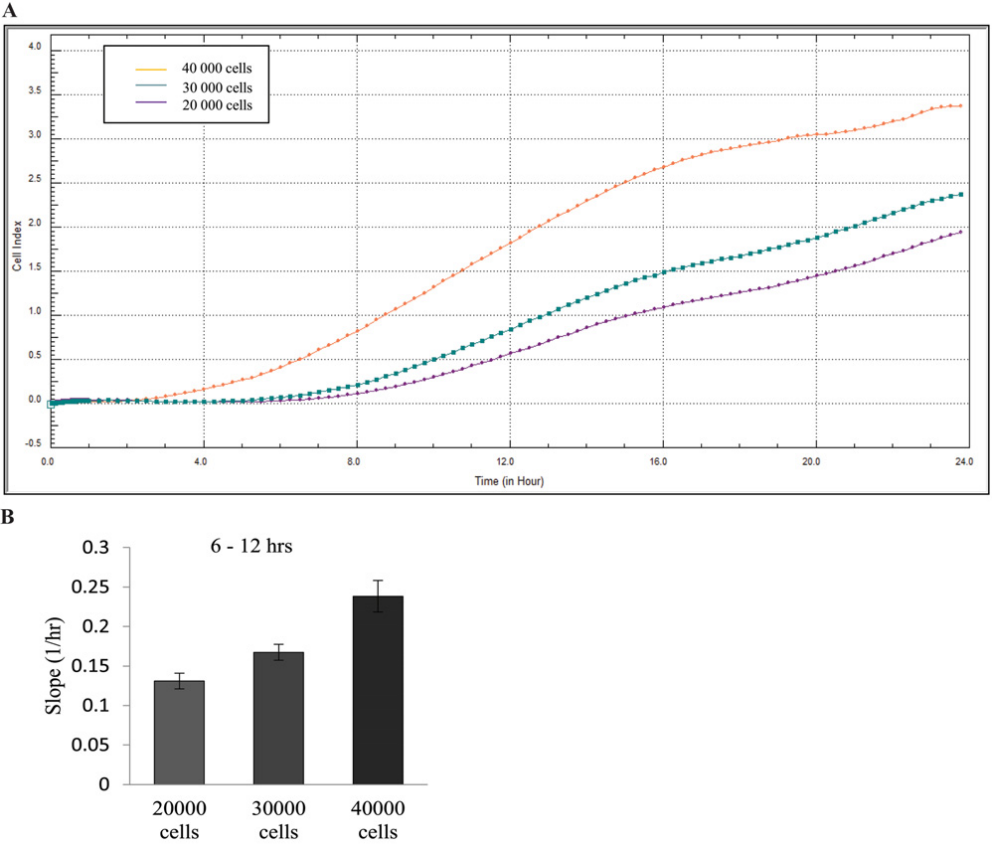
Optimizing cell number HCT116 cells were seeded at numbers ranging from 20 000, 30 000 and 40 000 in each well of an E-plate and the cells were automatically monitored every 30 seconds over 24 h. Results were expressed as a CI value. (**A**) Representative graph from xCELLigence system comparing the growth curve of HCT116 cells at 20 000 cells (purple line), 30 000 cells (green line) and 40 000 cells (orange line) (*n*=3). (**B**) Shown here is the rate of proliferation at the various cell concentrations as determined by analysing the slope of the line between the 6 and 12 h interval.

In this study, we were interested in using the RTCA platform to monitor how colon cancer cells behave in response to media derived from HDFs. First, it was necessary to determine the seeding concentration required to achieve a confluent monolayer of HCT116 cells. The cells were seeded at numbers ranging from 20 000 to 40 000 in each well of the E-plate and the cells were automatically monitored every 30 s over 24 h and expressed as a CI value (Figure 1A). Two distinct patterns can be seen on the graph, which can be attributed to cell adhesion and spreading (0–8 h) and cell proliferation (8–24 h). Our results also indicate that the rate of cell proliferation is dependent on cell confluency (Figure 1B). Based on these patterns, we determined that the optimum cell seeding density to monitor cell behaviour of HCT116 cells over 24 h is 40 000 cells/well.

### HDF media enhances cell adherence and proliferation

**Figure 2:**
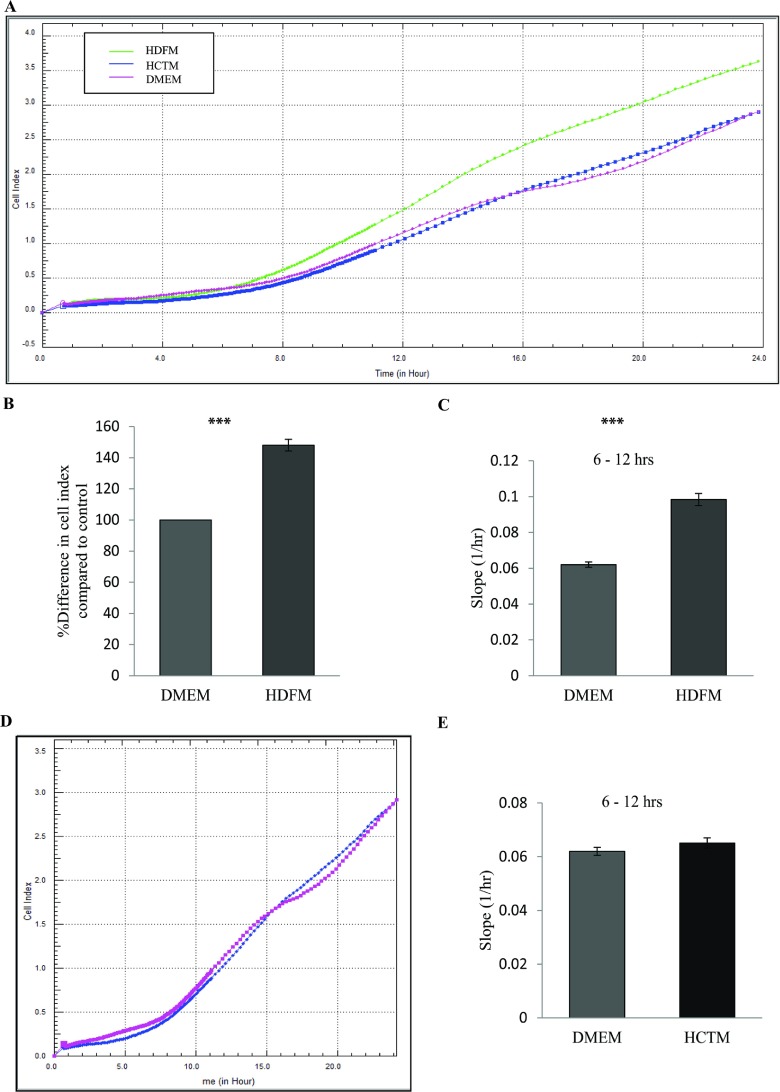
Effect of HDFM on the rate of proliferation of HCT116 cells HDFs were incubated with DMEM for 24 h. The media (HDFM) was removed and placed on HCT116 cells. The rate of proliferation was monitored in real-time using the xCELLligence system (*n*=3). (**A**) Representative graph comparing the rate of proliferation of HCT116 when incubated with HDFM (green line), HCTM (blue line) or DMEM (pink line). (**B**) Comparison of the percentage difference in the mean CI between the cells incubated with HDFM or DMEM. (*P*<0.05, *n*=3). (**C**) The rate of proliferation as determined by analysing the slope of the line between the 6 and 12 hour interval (*P*<0.05, *n*=3). (**D**) Representative graph comparing the rate of proliferation of HCT116 when incubated with the two controls HCTM (blue line) or DMEM (pink line). (**E**) The rate of proliferation of the controls as compared between the 6 and 12 h interval.

**Figure 3:**
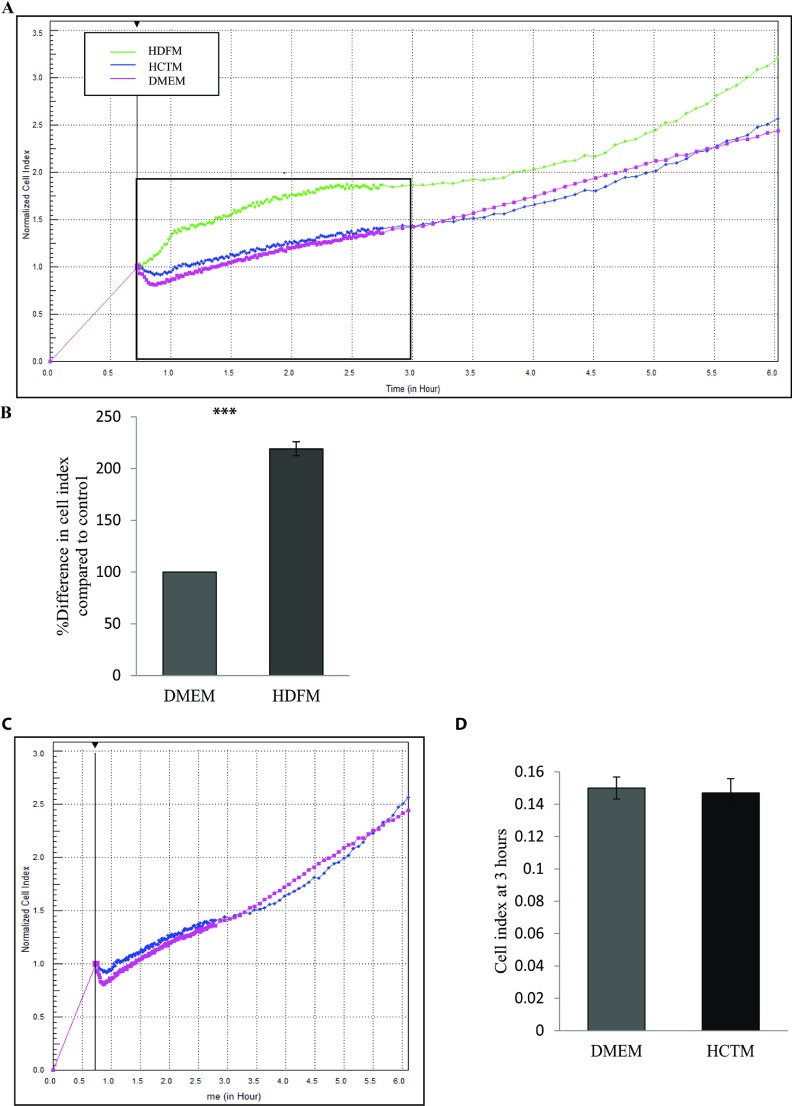
Effect of HDFM on the rate of adherence of HCT116 cells HDFs were incubated with DMEM for 24 h. The media (HDFM) was removed and placed on HCT116 cells. The rate of adherence was monitored between 0 and 3 h, in real-time using the xCELLligence system (*n*=3). (**A**) Representative graph comparing the rate of adherence of HCT116 when incubated with HDFM (green line), HCTM (blue line) or DMEM (pink line). (**B**) Comparison of the percentage difference in the mean CI between the cells incubated with HDFM or DMEM (*P*<0.05, *n*=3). (**C**) Representative graph comparing the rate of adherence of HCT116 when incubated with the two controls HCTM (blue line) or DMEM (pink line). (**D**) The CI of the two controls measured at 3 h.

Having determined the optimal conditions to study the behaviour of colon cancer cells, we next wanted to determine the effect of culturing HCT116 cells in the presence of media derived from HDFs. To do this, HCT116 cells were seeded in the presence of media taken from HDF cultures [HDFM (human dermal fibroblast medium)] (see the Methods section) and compared with HCT116 cells that were seeded in the presence of DMEM and control HCTM (media derived from HCT116 cultures). Cell behaviour was monitored using RTCA over a period of 72 h with data shown for the first 24 h (Figure 2A and Supplementary Figure S1A). Results indicate that HCT116 cells proliferate significantly faster when grown in the presence of media derived from HDFs (*P*<0.05, *n*=3) (Figures 2B and 2C, and Supplementary Figure S2A). Importantly, when HCT116 cells were grown in the presence of DMEM or DMEM derived from HCT116 cultures, there was no difference in growth patterns and cell behaviour [Figures 2D and 2E and Supplementary Figure S2B (available at http://www.bioscirep.org/bsr/034/bsr034e126add.htm)]. These data suggest that HDFs drive the transformed phenotype in colon cancer. To investigate the effects on cell adhesion, data were extracted from the platform over the first 3 h of cell monitoring. Data were normalized at 40 min to allow for any discrepancy in CI as the cells settle. The results show that HCT116 cells incubated with media derived from HDF cells adhere more than twice as fast as the controls (*P*<0.05, *n*=3) (Figure 3A and 3B, and Supplementary Figure S1B). As above, this effect was not seen when HCT116 cells were incubated in the presence of media derived from HCT116 cultures (Figures 3C and 3D and Supplementary Figure S1B).

**Figure 4:**
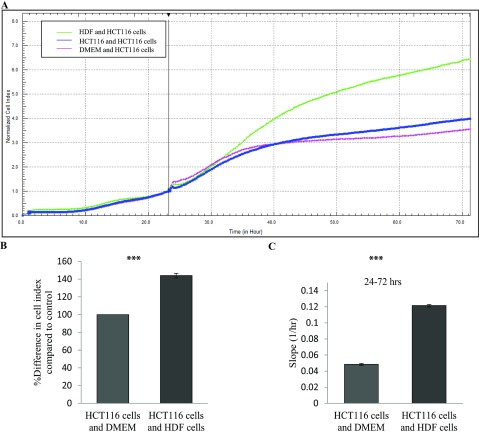
Effect of co-culturing HDF cells with HCT116 cells HCT116 cells were seeded in the E-plate 16. 24 h later an E-plate insert containing HDF cells was placed into the E-plate 16. The rate of proliferation was monitored in real-time using the xCELLigence system. (**A**) Representative graph comparing the rate of proliferation of HCT116 co-cultured with HDF cells (green line), HCT116 cells (blue line) or DMEM (pink line). (**B**) Comparison of the percentage difference in the mean CI between the cells co cultured with HDF cells or DMEM (*P*<0.05, n=1). (**C**) The rate of proliferation as determined by analysing the slope of the line between the 24 and 72 h interval (*P*<0.05).

Next, we looked at the effect of co-culturing HDF cells with HCT116 cells. To execute this, HCT116 cells were seeded in an E-plate 16. 24 h later an E-plate insert containing live cultures of HDF cells were placed into the E-plate 16 and the rate of proliferation was monitored in real-time using the xCELLigence system. As expected, the HCT116 cells proliferated much faster when co-cultured with HDF in comparison with controls (Figure 4) (*P*<0.05). Taken together, this strongly supports the hypothesis that fibroblasts drive cancer progression.

### Colon cancer cells migrate and invade faster in the presence of media derived from HDFs

**Figure 5:**
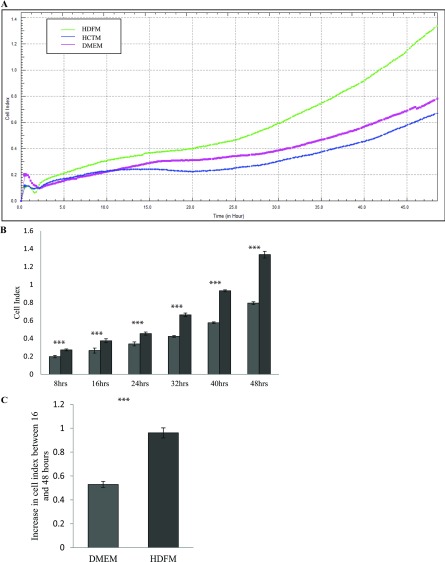
Effect of HDFM on the rate of migration of HCT116 cells HDF cells were incubated with DMEM for 24 h. The media (HDFM) was removed and placed in the LC of the CIM -plate with HCT116 cells in the UC (see the Method section). The rate of migration was monitored in real-time using the xCELLigence system (*n*=3). (**A)** Representative graph comparing the rate of migration of HCT116 towards HDFM (green line), HCTM (blue line) or DMEM (pink line). (**B**) Comparison of the CI between the cells migrating towards HDFM or DMEM, at 8, 16, 24, 32, 40 and 48 h (*P*<0.05, *n*=3). (**C**) The change in CI between 16 and 48 h (*P*<0.05, *n*=3).

Following this, we wanted to investigate if media derived from HDFs promotes the migration and invasion of HCT116 cells. The effect of HDF media on the migration of HCT116 cells was monitored using the xCELLigence CIM-plates configuration, similar in design to a Boyden Chamber but with built in RTCA capabilities (see the Methods section). HCT116 cells were placed in the UCs of the CIM-plates, whereas media derived from HDFs was placed in the LC. The migration of HCT116 cells from the UC to the media in the LC was monitored over 48 h as the cells passed through the electrodes and fibronectin coating (Figures 5A, 5B and 5C and Supplementary Figure S1C). The results indicate that HCT116 cells migrate almost twice as fast towards HDF media when compared with the controls (*P*<0.05, *n*=3).

**Figure 6:**
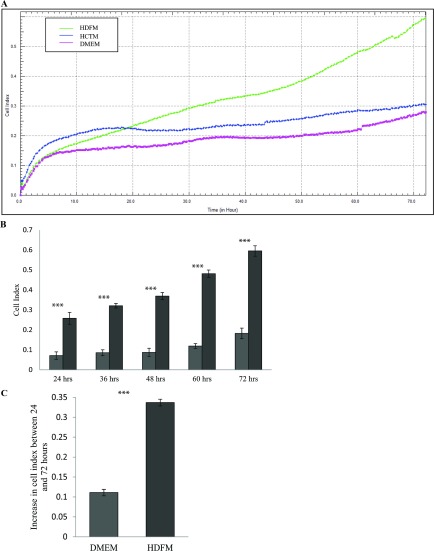
Effect of HDFM on the rate of invasion of HCT116 cells HDF cells were incubated DMEM for 24 h. The media (HDFM) was removed and placed in the LC of the CIM-plate with HCT116 cells in the UC. The UC of the CIM-plate was coated in matrigel. The rate of invasion was monitored in real-time using the xCELLigence system (*n*=3). (**A**) Representative graph comparing the rate of invasion of HCT116 cells as they move towards HDFM (green line), HCTM (blue line) or DMEM (pink line). (**B**) Comparison of CI between the cells invading the matrigel layer towards HDFM or DMEM, at 24, 36, 48, 60 and 72 h (*P*<0.05, *n*=3). (**C**) The change in CI between 24 and 72 h (*P*<0.05, *n*=3).

To test whether HDFs can promote colon cancer cell invasion, we employed a 3D invasion model. To execute this, we set up the experiments as above, with a coating of fibronectin and a layer of matrigel added to the CIM-plates to simulate the ECM and to provide a barrier through which the cells would have to invade. HCT116 cells were placed in the UC of the CIM-plates, on top of the pre-formed matrigel layer. As above, media derived from HDFs was placed in the LC and the cells were monitored over 72 h. Only HCT116 cells that invaded the matrigel layer, and passed through the UC to the LC were detected by the electrodes built into the CIM-plates. A minor change in CI was recorded when HCT116 cells were invading towards DMEM or media derived from HCT116 cultures indicating that little or no invasion and directional cell migration through the matrigel layer occurred. In contrast to this, HCT116 cells demonstrated a strong invasive phenotype when media derived from HDFs was placed in the LC of the CIM-plates. This indicated that media derived from HDFs provide an attractive gradient for HCT116 cells and provides strong evidence that HDFs promote the migratory and invasive phenotype of colon cancer cells (*P*<0.05, *n*=3) (Figures 6A, 6B and 6C and Supplementary Figure S1D).

## DISCUSSION

Monitoring how cancer cells respond to changes in the stromal environment is a fundamental question in biomedical research [1,14,34,35] but is hindered by an overreliance on end-point assays. These assays do not facilitate continuous monitoring of cell adhesion, proliferation, migration and invasion, the monitoring of which is vital in determining the effect of the cells external environment. In this study, we used RTCA technology (xCELLigence RTCA DP, ACEA) to provide real-time data on how fibroblasts alter the behaviour of colon cancer cells. Here, we have established that media derived from human fibroblasts can drive the transformed phenotype in cancer by significantly increasing the proliferation, migration and directional invasion kinetics of colon cancer cells.

The xCELLigence RTCA platform is proving to be a highly accurate platform to monitor cell behaviour [32,33,36–38] and it correlates very well with conventional adhesion, migration and invasion assays [39]. As well as this, the non-invasive and label-free platform is being used as a tool for high-throughput drug screening and as a robust system to measure the toxicological response to nanoparticles and novel compounds [40–42].

In this study, we used E-plates and CIM-plates for all experiments, which were carried out on an xCELLigence DP system. E-plates containing 16 wells in a standard microtitre plate format are used for adhesion and proliferation assays. CIM-plates are similar to a modified Boyden Chamber and can be used to monitor cell migration and invasion in real-time. Both plates facilitate the simultaneous monitoring of several experiments with minimal user input and have made it possible to obtain real-time data on changes in cell adhesion, proliferation, migration and invasion. This system has great potential for reducing and refining traditional experimental procedures. As the xCELLigence system is an impedance-based platform, the changes in CI values can be interpreted as morphological changes in the cells, with distinct patterns emerging when several replicate and control experiments are run in parallel.

We first established the optimum growth of HCT116 cells on our system, using the slope of the growth curve we determined there was a direct relationship between the CI and cell numbers (Figures 1A and 1B). This allowed us to choose 40 000 cells/well as the optimal cell number to perform our experiments and monitor the effects of fibroblast on tumour cell growth. When we exposed HCT116 cells to media taken from HDFs, we show that there was a significant increase in CI, which correlates with increased cell proliferation (Figures 2A and 2B). To further emphasize this, we calculated the slope of the growth curve (Figure 2C), which we found to be much greater at all 6-h intervals in the experiment (results shown for 6–12 h interval). We included two controls in our experiments. The first was culturing HCT116 cells in the presence of fresh DMEM with 10% (v/v) FBS and the second was culturing HCT116 cells in media that was taken from 24 h cultures of other HCT116 cells (see the Methods section). These controls were informative as they allowed us to conclude that any changes we were seeing in colon cell cultures in the presence of media derived from HDFs was because of the factors released from the fibroblasts. Had these controls not been included one could have argued that changes we were observing was due to the changes in the cell culture media as a result of cell exposure for 24 h. Using xCELLigence growth curves to calculate the slope of the growth curve (Figures 2D and 2E), we show that there was no difference between the two controls and both were carried through all experiments.

As the xCELLigence technology measures impedance changes across the meshwork of microelectrodes on the bottom of an E-plate, the gradual occupation of cells on the electrode surface alters the impedance of the electrodes [26,27,39]. In the initial 3-4 h of an experiment, we can interpret the CI change as data on cell adhesion and cell spreading when controlled closely by parallel phase contrast microscopy (Supplementary Figures S3A and S3B, available at http://www.bioscirep.org/bsr/034/bsr034e126add.htm). When HCT116 cells were grown in the presence of media derived from HDFs, there was a dramatic increase in the rate of cell adhesion to the microelectrodes suggesting that factors secreted by fibroblasts increase the cell adhesion kinetics of the cancer cells (Figures 3A and 3B). To emphasize this, using E-plate inserts (Figure 4), we showed that direct co-culture of HCT116 and HDFs increased HCT116 cell proliferation.

Using the CIM-plates, which have a series of interdigitated microelectrodes on the bottom side of a microporous membrane, we were able to determine the kinetics by which the HCT116 cells migrate in the presence of media derived from HDFs. Data from our chemotactically driven migration setup indicate that the cancer cells migrated much faster through the microporous membrane in chambers containing media derived from HDFs rather than control media (Figures 5A–5C). In these experiments, the RTCA platform facilitates a much more accurate detection of the migration process as it records CI changes as the cells pass through the electrode in real-time. This is more advantageous then the traditional assays of staining and counting cells on the bottom of a modified Boyden Chamber.

We then carried out a series of experiments to determine if the media derived from HDFs would promote the invasion of HCT116 cells (Figure 6). Again, our experimental design was based on the xCELLigence platform with the CIM-plates except this time, we challenged the cells to invade a matrigel layer while migrating towards the same chemotactic gradient as described above. Our results were quiet dramatic and suggested that media derived from HDFs not only drive colon cancer cell migration, but also increase their invasive capacity. Again, these data are much more reliable quantitation of cell invasion when compared with OD measurements (crystal violet) and cell number/area calculations done on the bottom of transwell membranes [43,44]. In particular, our migration and invasion profiles were highly reliable and very reproducible and the platform supports a higher sensitivity compared with traditional end-point assays. As well as this, the system is more immune from intra-individual variation regarding cell culture handling. The reliability and reproducibility of our migration and invasion experiments indicate that the xCELLigence RTCA platform performs well in comparison with the classic detection methods.

How might fibroblasts be regulating cancer cell growth? The stromal environment can influence tumourigenesis and can promote angiogenesis and metastasis. Fibroblasts, as a central component of the stromal environment, can influence the composition of the ECM [14] and regulate growth factor availability and presentation by secreting growth factors and growth factor-binding proteins. There are several reports indicating that fibroblasts in the stromal tissue lay down ECM proteins that are suggested routes of cancer cell metastasis [1,2,45,46]. Our migration and invasion data also suggest that fibroblasts release soluble factors that attract cancer cells and should be considered more widely as therapeutic targets in cancer. Subtle changes in the composition of the ECM can have dramatic effects on cancer cell behaviour. Attachment of cells to the ECM is mediated by focal adhesions, (reviewed in [47]). Focal adhesions are large dynamic macromolecular protein complexes commonly found at the leading edge of migrating cells and are the mechanical link between the cell and the ECM, formed after integrins are clustered on the cell surface in response to signals from the ECM. Integrin clustering is sufficient to promote the phosphorylation of FAK (focal adhesion kinase), which correlates with an increase in kinase activity and the ability of FAK to cooperate with multiple signalling pathways through its direct interaction with other non-receptor tyrosine kinases, cell surface receptors, cytoskeletal proteins and other adaptor proteins. FAK is widely regarded as being the convergence point of growth factor and integrin signalling and FAK regulates cell adhesion and cell migration in response to growth factors and integrin stimulation and in response to changes in the composition of the ECM [48–50]. FAK activity is heavily influenced by growth factors and even subtle changes in FAK activity, or in the expression patterns of the proteins central to focal adhesions, will remodel focal adhesions to promote cell migration and invasion of cancer cells [51]. Further studies that characterize the specific factors secreted by HDFs in our system and similar co-culture systems will be very beneficial to the wider community and is a focus of this research group.

This is the first study of its kind that uses RTCA platforms to show that media derived from HDFs promotes the transformed phenotype in cancer. We used the RTCA platform to show that HDF media increased adhesion, proliferation, migration and invasion of colon cancer cells. In our opinion, RTCA platforms would be a robust and reliable technology to assess the kinetic screening of novel compounds that alter cell morphology and migration/invasion dynamics.

## Online data

Supplementary data
