# Supplementary material for: Using real-time impedance-based assays to monitor the effects of fibroblast-derived media on the adhesion, proliferation, migration and invasion of colon cancer cells
Source: Biosci Rep. 2014 Jul 29;34(4):e00126. doi: 10.1042/BSR20140031 (PMC4114067; doi:10.1042/BSR20140031)
Supplement: Supplementary data [file bsr034e126add.pdf]

Biosci. Rep. (2014) / **34** / art:e00126 / doi 10.1042/BSR20140031

OPEN ACCESS

Original Paper

SUPPLEMENTARY DATA

Using real-time impedance-based assays to monitor the effects of fibroblast-derived media on the adhesion, proliferation, migration and invasion of colon cancer cells

Catríona M. DOWLING*†, Carmen HERRANZ ORS* and Patrick A. KIELY*†[1]

*Department of Life Sciences, and Materials and Surface Science Institute, University of Limerick, Limerick, Ireland
†Stokes Institute, University of Limerick, Limerick, Ireland

Bioscience Reports

www.bioscirep.org

Bioscience Reports

Supplementary Figures S1–S3 are on the following pages.

[1] To whom correspondence should be addressed (email Patrick.Kiely@ul.ie).

© 2014 The Author(s) This is an Open Access article distributed under the terms of the Creative Commons Attribution Licence (CC-BY) (http://creativecommons.org/licenses/by/3.0/) which permits unrestricted use, distribution and reproduction in any medium, provided the original work is properly cited.

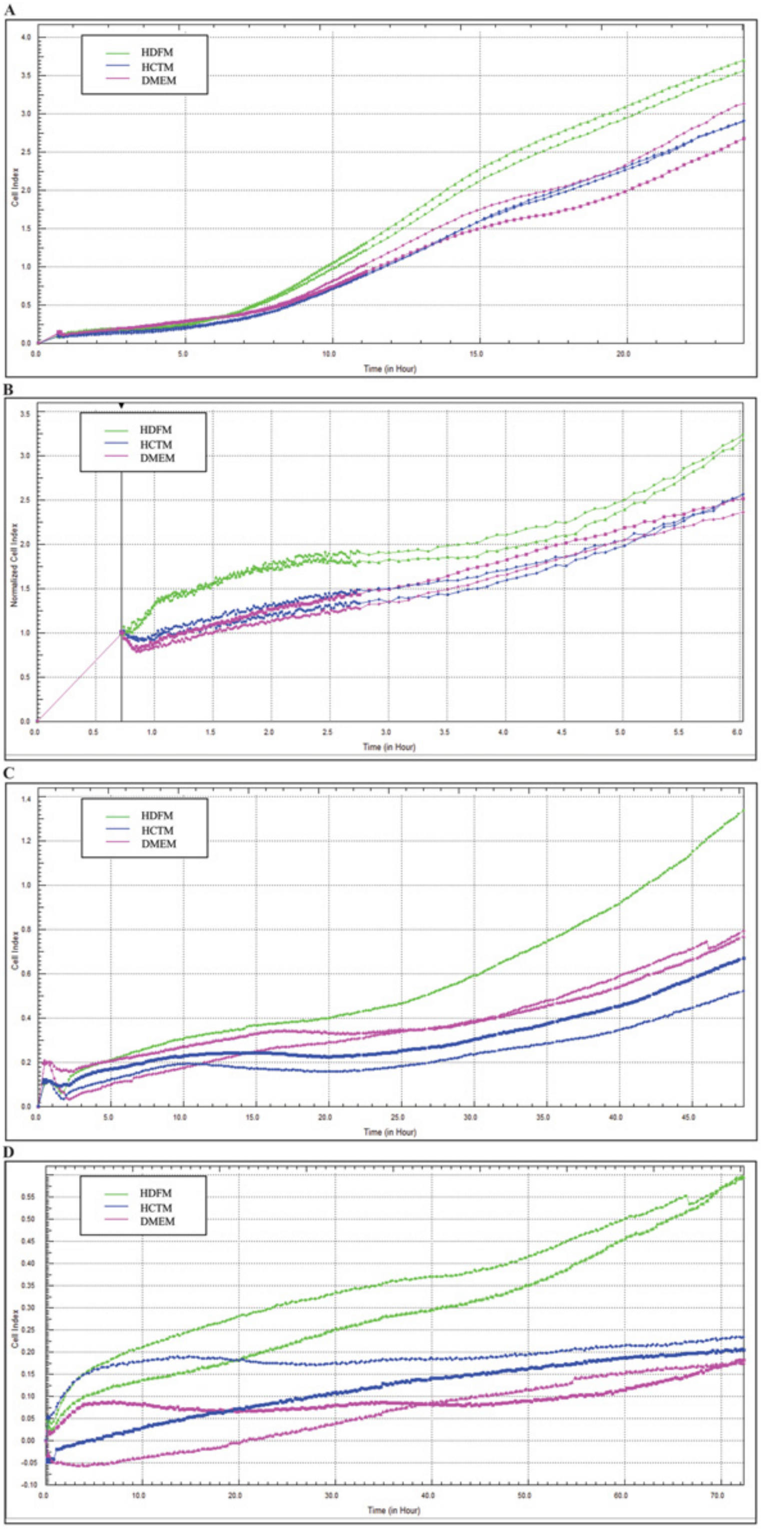

Figure S1 Technical replicates showing no significant intra-experimental variability

All experiments were conducted three times and each time the experiment was done in duplicate wells. (**A**) Technical replicates for proliferation experiment. (**B**) Technical replicates for adhesion experiment. (**C**) Technical replicates for migration experiment. (**D**) Technical replicates for invasion experiment.

© 2014 The Author(s) This is an Open Access article distributed under the terms of the Creative Commons Attribution Licence (CC-BY) (http://creativecommons.org/licenses/by/3.0/) which permits unrestricted use, distribution and reproduction in any medium, provided the original work is properly cited.

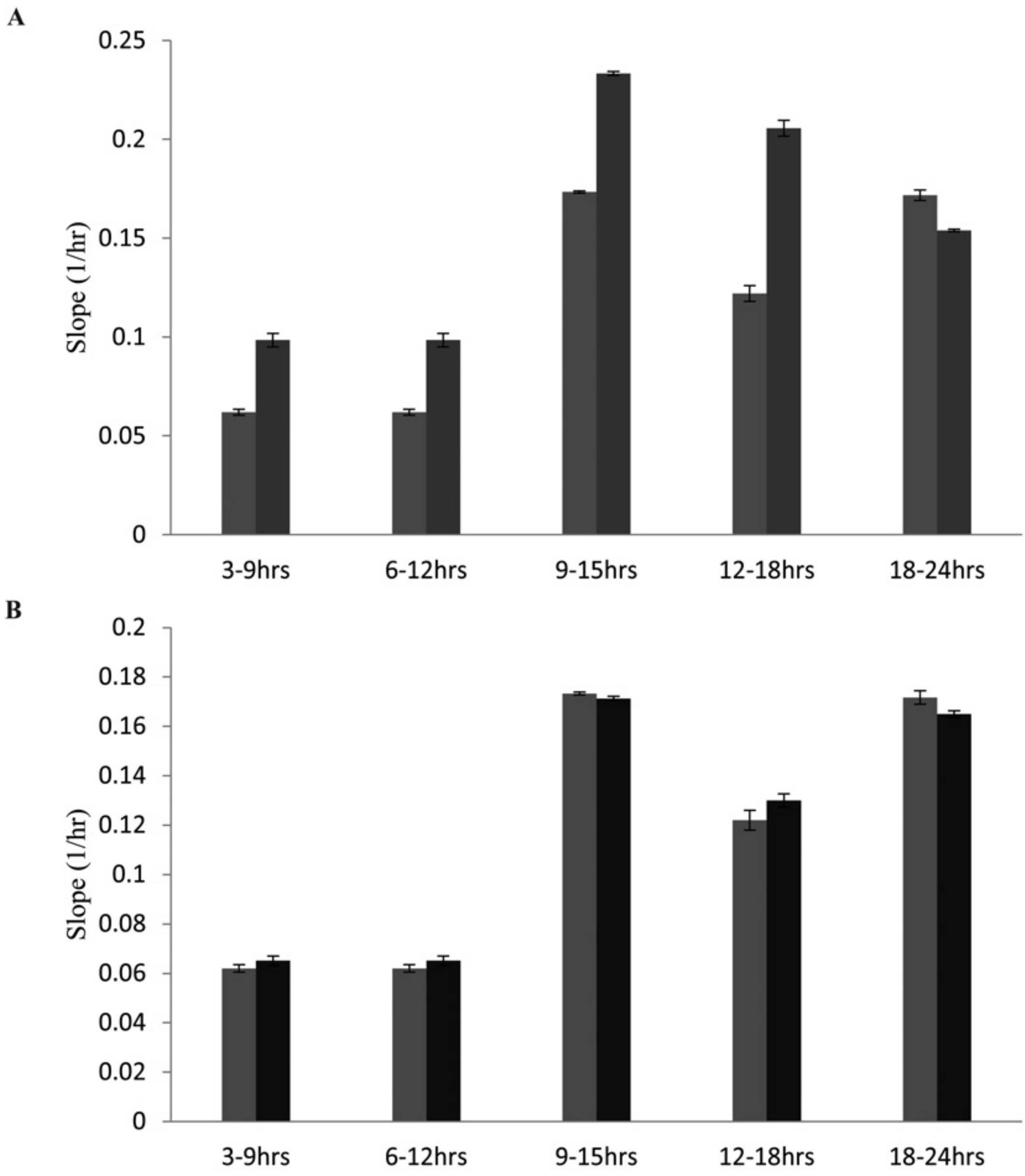

Figure S2 **Effect of HDFM on the rate of proliferation and adherence of HCT116 cells**
The rate of proliferation (**A**) and adherence (**B**) as determined by analysing the slope of the line between five different 6 h intervals ($n = 3$).

© 2014 The Author(s) This is an Open Access article distributed under the terms of the Creative Commons Attribution Licence (CC-BY) (http://creativecommons.org/licenses/by/3.0/) which permits unrestricted use, distribution and reproduction in any medium, provided the original work is properly cited.

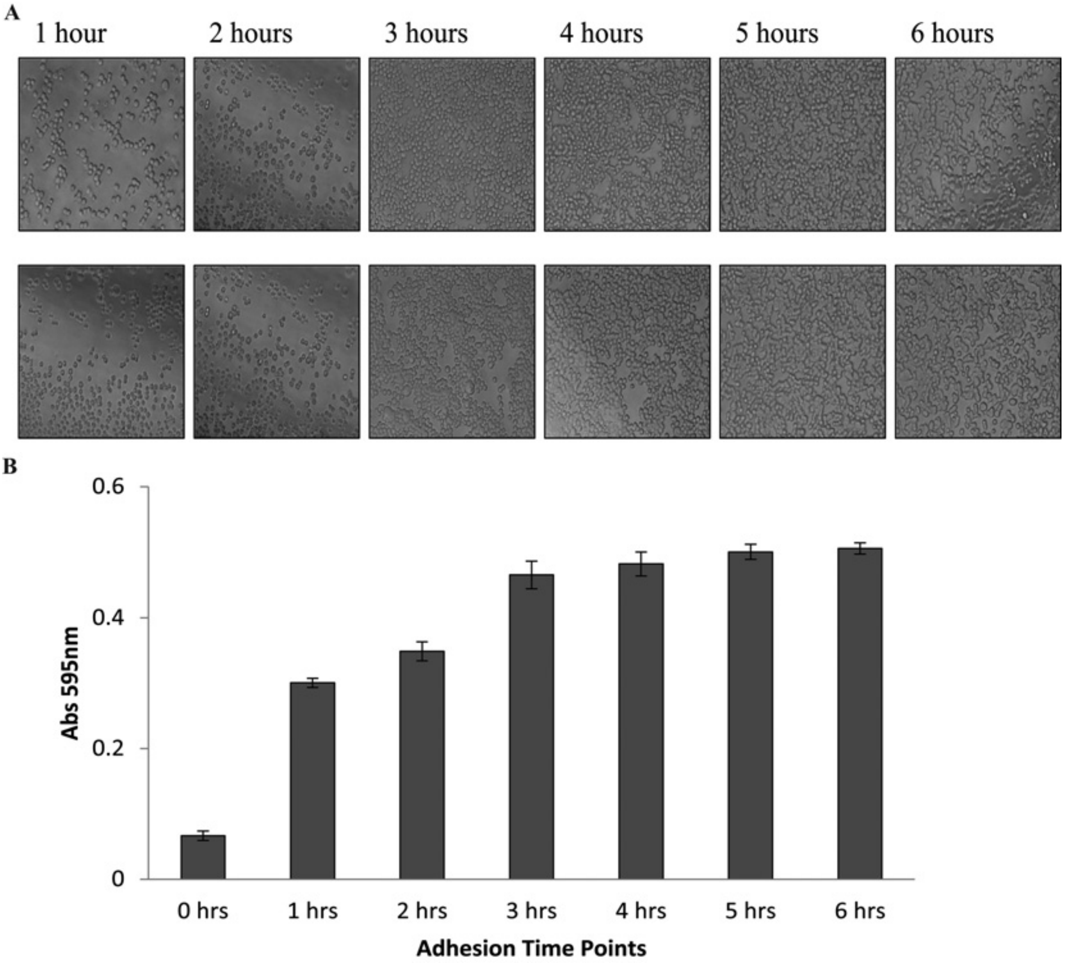

Figure S3 **RTCA data correlates well with traditional adhesion assays**
HCT116 cells were seeded in a 96-well plate; adherence was monitored using cell microscopy and crystal violet staining every hour for 6 h. (**A**) Live cell microscopy of HCT116 cells over a period of 6 h. (**B**) Data for crystal violet stained cells, absorbance were determined at 595 nm over a period of 6 h.

Received 21 February 2014/30 April 2014; accepted 7 May 2014

Published as Immediate Publication 17 June 2014, doi 10.1042/BSR20140031

© 2014 The Author(s) This is an Open Access article distributed under the terms of the Creative Commons Attribution Licence (CC-BY) (http://creativecommons.org/licenses/by/3.0/) which permits unrestricted use, distribution and reproduction in any medium, provided the original work is properly cited.
